# miR-4284 Functions as a Tumor Suppressor in Renal Cell Carcinoma Cells by Targeting Glutamate Decarboxylase 1

**DOI:** 10.3390/cancers15153888

**Published:** 2023-07-30

**Authors:** Sujin Choi, Kyeongmi Kim, Hyunjeong Yeo, Gyurim Lee, Isaac Kim, Jisu Oh, Hyun-Ju An, Soonchul Lee

**Affiliations:** 1 Department of Orthopaedic Surgery, CHA Bundang Medical Center, CHA University School of Medicine, 335 Pangyo-ro, Bundang-gu, Seongnam-si 13488, Gyeonggi-do, Republic of Korea; bsujin35@naver.com (S.C.); lallalla106@naver.com (H.Y.); kr95labs@naver.com (G.L.); 2 Department of Laboratory Medicine, CHA Ilsan Medical Center, CHA University School of Medicine, 1205, Jungang-ro, Ilsandong-gu, Goyang-si 10414, Gyeonggi-do, Republic of Korea; kmi0905@chamc.co.kr; 3 Department of General Surgery, CHA Bundang Medical Center, CHA University School of Medicine, 335 Pangyo-ro, Bundang-gu, Seongnam-si 13488, Gyeonggi-do, Republic of Korea; 24icecream@hanmail.net; 4 Division of Hemato-Oncology, Department of Internal Medicine, Yongin Severance Hospital, Yonsei University College of Medicine, 363 Dongbaekjukjeon-daero, Giheung-gu, Seoul 16995, Gyeonggi-do, Republic of Korea; newfascia@gmail.com; 5 SL Bio, Inc., 120 Haeryong-ro, Pocheon-si 11160, Gyeonggi-do, Republic of Korea

**Keywords:** microRNA, miR-4284, renal cell carcinoma, glutamate decarboxylase 1, tumor suppressor

## Abstract

**Simple Summary:**

miRNAs play a crucial role as oncogenic or tumor suppressors in the pathogenesis and progression of tumors. However, few studies have investigated the exact role of miR-4284 in RCC. Thus, we investigated the role of miR-4284 as a tumor suppressor in renal cancer cell lines. In this study, miR-4284 overexpression suppressed proliferation, induced apoptosis, and suppressed tumorigenic features of renal cancer cells. GAD1 was directly targeted by miR-4284. A xenograft mouse model injected with Caki-1 cells transfected with miR-4284 showed significantly decreased tumor growth rate and volume. Our study provided novel findings about the miR-4284 functions as a tumor suppressor in RCC by targeting GAD-1. These findings highlight the potential of miR-4284 as a target for anticancer miRNA therapeutics in RCC.

**Abstract:**

MicroRNAs (miRNAs) play a crucial role as oncogenic or tumor suppressors in the pathogenesis and progression of tumors. However, few studies have investigated the exact role of miR-4284 in renal cell carcinoma (RCC). We aimed to investigate the role of miR-4284 as a tumor suppressor in renal cancer cell lines. A498 and Caki-1 were transfected with miR-4284. The Cell Counting Kit-8, colony formation, apoptosis assays, and quantitative reverse transcription–polymerase chain reaction were used to evaluate tumor growth-inhibiting functions. The wound-healing, transwell, and sphere-formation assays were conducted to investigate tumorigenic characteristics. The potential target genes of miR-4284 were predicted and experimentally verified. A xenograft experiment was performed to estimate the tumor-growth-suppressive function of miR-4284. miR-4284 overexpression suppressed proliferation, induced apoptosis, and suppressed tumorigenic features of renal cancer cells. Glutamate decarboxylase 1 (GAD1) was directly targeted by miR-4284. A xenograft mouse model injected with Caki-1 cells transfected with miR-4284 showed significantly decreased tumor growth rate and volume. miR-4284 affected tumor growth, metastasis, and apoptosis of renal cancer cells in vitro and in vivo. These findings highlight the potential of miR-4284 as a target for anticancer miRNA therapeutics in RCC.

## 1. Introduction

According to the World Health Organization, cancer is a leading cause of mortality and morbidity worldwide, with approximately 19.3 million new diagnoses and almost 10.0 million deaths in 2020 [1]. Renal cell carcinoma (RCC) accounts for 2–3% of adult malignant tumors and has limited treatment options, such as cryotherapy and radiofrequency ablation therapy, except for surgery and stereotactic body radiation therapy [2]. At the time of diagnosis, approximately 17% of RCCs are locally advanced, and 17% have distant metastasis [3]. However, currently, there is no reliable biological marker for the early detection of RCC, and RCC is frequently discovered incidentally on radiological examinations, such as computed tomography or ultrasonography.

MicroRNAs (miRNAs) are small noncoding RNA molecules composed of 21–23 nucleotides that are involved in RNA silencing and gene expression regulation during posttranscriptional processing [4]. An miRNA called lin-4, which regulates the developmental stage of *Caenorhabditis elegans*, was discovered for the first time by Lee et al. [5]. In addition, Calin et al. confirmed that miRNAs (miR-15a and miR-16-1) acted as tumor suppressors in B-cell chronic lymphoid leukemia cells [6]. miRNAs are involved in various biological processes, including oncogenesis and metastasis. Recently, several miRNAs have been proposed as potential biological factors for the diagnosis, prognosis, and treatment of cancer, and several studies have been conducted regarding this.

In RCC, several miRNAs have been suggested as potential factors that affect pathophysiology, diagnosis, prognosis, and treatment targets. For example, miR-210 or miR-200 family members are involved in key pathogenesis mechanisms, such as hypoxia (hypoxia-inducible factor 1/von Hippel–Lindau (VHL)-dependent) and epithelial-to-mesenchymal transition in RCC [7,8,9,10]. miR-210, miR-21, and miR-155 are upregulated, whereas miR-141, mir-200 family, and mir-138 are downregulated, suggesting the possibility of aiding the diagnosis of RCC [11,12,13]. Studies have been published stating that miR-4284, one of the miR-RNAs, affects cancer cells by being upregulated in non-small-cell lung, gastric, and ovarian cancer cells [14,15,16]. However, few studies have investigated the exact role of miR-4284 in RCC.

In this study, we aimed to investigate the potential role of miR-4284 as a tumor suppressor in RCC and to identify the direct target molecules involved in its action. In addition, we analyzed the effect of miR-4284 on tumor growth in a mouse xenograft model.

## 2. Materials and Methods

### 2.1. Cells and Animals

The human renal cancer cell lines A498 and Caki-1 were obtained from the Korean Cell Line Bank. A498 and Caki-1 cells were cultured in Roswell Park Memorial Institute-1640 medium (WELGENE, Daegu, Republic of Korea) supplemented with 10% fetal bovine serum (Gibco; Thermo Fisher Scientific, Carlsbad, CA, USA) and 1% penicillin–streptomycin (Gibco). The cells were maintained in an incubator conditioned with 5% CO_2_ at 37 °C. Four-week-old female BALB/c nu/nu mice were purchased from ORIENT BIO. Animals were maintained at 23 ± 1 °C and 50 ± 10% humidity under specific pathogen-free conditions. The light–dark period was cycled every 12 h, and food and water were provided ad libitum. Animal experimental procedures were reviewed and approved by the CHA University Animal Care and Use Committee (IACUC210152).

### 2.2. miRNA Mimics and Plasmid Vector Transfection

G-fectin (Gelolution Pharmaceuticals Co., Seoul, Republic of Korea) was used as an miRNA transfection reagent for transfection with miRNA mimics. According to the manufacturer’s protocol, cells were seeded at 30% confluency with a medium containing G-fectin and miRNA mimic mixture and incubated for 48 h. For transfection with plasmid vectors, Lipofectamine 3000 (L3000015, Thermo Fisher Scientific) was used as a plasmid vector transfection reagent. According to the manufacturer’s protocol, cells were seeded at 70% confluency and incubated for 24 h, changed to the medium containing Lipofectamine 3000 and plasmid vector mixture, and incubated for 48 h.

#### 2.2.1. Real-Time Reverse Transcription-PCR Analysis of mRNA

To prepare the total RNA, A498 and Caki-1 cells were lysed using TRIzol Reagent (Invitrogen, Thermo Fisher Scientific) after transfection with miRNA mimics. Total RNA was synthesized into cDNA using the Maxime RT PreMix Kit (iNtRON, Seongnam, Republic of Korea). In brief, 1 μg of total RNA and pure water were added into the Maxime RT PreMix tubes up to 20 μL, and the mixture was incubated as follows, according to the manufacturer’s protocol: cDNA synthesis, 45 °C for 60 min, and RTase inactivation step, 95 °C for 5 min. To quantify mRNA expression, qRT-PCR analysis was performed using the AMPIGENE qPCR Green Mix Lo-ROX (Enzo Life Sciences, Seoul, Republic of Korea). According to the manufacturer’s protocol, a mixture of cDNA, reagents, and specific primers was prepared and incubated as follows: one cycle, 95 °C for 2 min; 40 cycles, 95 °C for 5 s; and 60 °C for 30 s. The specific primers used are listed in Table 1. Data were analyzed using the 2^−ΔΔCt^ method and normalized to glyceraldehyde-3-phosphate dehydrogenase expression.

#### 2.2.2. Real-Time Reverse Transcription-PCR Analysis of miRNA

After preparing the total RNA, the total RNA was converted to cDNA using HB_I RT Reaction kit (HeimBiotek, Seongnam, Republic of Korea). In brief, a mixture of 1 μg of total RNA and reagents was prepared and incubated as follows, according to the manufacturer’s protocol: incubation, 37 °C for 60 min; inactivation, 95 °C for 5 min. To quantify the miRNA expression, qRT-PCR analysis was performed using HB_I Real-Time PCR Master Mix kit (HeimBiotek, Seongnam, Republic of Korea). According to the manufacturer’s protocol, a mixture of cDNA, reagents, and specific primers was prepared into the tube up to a total volume of 20 μL and incubated as follows for the reaction: one cycle, 95 °C for 15 min; 40 cycles, 95 °C for 10 s; and 60 °C for 40 s. The specific primers were as follows: miR-4284 and 5′- GGG CUC ACA UCA CCC CAU -3′. Data were analyzed using the 2^−ΔΔCt^ method and normalized to RNA, U6 small nuclear 6, and pseudogene expression.

### 2.3. Cell Viability Assay

To estimate the viability of miR-4284 in human renal cancer cells, a cell viability assay was performed using the CCK-8 assay (Dojindo, Kumamoto, Japan). A498 and Caki-1 cells were seeded in a 96-well plate and transfected with miRNA mimics for 48 h. Subsequently, 10 μL of CCK-8 solution was added to each well, and the absorbance was detected at 450 nm wavelength after incubation for 2 h.

### 2.4. Colony-Forming Assay

The proliferation ability of miR-4284 in human renal cancer cells was estimated using a colony-forming assay. Briefly, A498 and Caki-1 cells were transfected with 40 nM miRNA mimics for 48 h. The transfected cells were then reseeded to a 60 mm dish with 500 cells/dish and incubated, with a medium change every 2–3 days until colony formation. Crystal violet staining was performed to visualize colonies. The cells were washed once with phosphate-buffered saline (PBS), fixed with 3.7% formaldehyde in PBS for 10 min, incubated with 0.05% crystal violet solution (Sigma Aldrich, Burlington, MA, USA) in 10% ethanol for 30 min, rinsed twice with distilled water, dried, and counted.

### 2.5. Apoptotic Cell Analysis

The apoptotic function of miR-4284 in human renal cancer cells was examined using the annexin V/PI apoptosis assay. Briefly, A498 and Caki-1 cells were transfected with 40 nM miRNA mimics for 48 h, collected, washed once with PBS, and resuspended to a 1X binding buffer. The resuspended cells were mixed with 5 μL of annexin V-fluorescein isothiocyante and PI solution and incubated for 15 min. The stained cells were analyzed using a CytoFLEX Flow Cytometer (Beckman Coulter, Brea, CA, USA) and CytExpert software version 2.4 (Beckman Coulter, Brea, CA, USA).

### 2.6. Wound-Healing Assay

A wound-healing assay was conducted to examine cell migration ability. A498 and Caki-1 cells were transfected with 40 nM miRNA mimics for 48 h and wounded using a pipette tip. The cells were incubated, and the indicated times were captured using a Nikon Eclipse Ts2-FL Diascopic and Epi-fluorescence illumination model. The wound area was measured using ImageJ 1.52a.

### 2.7. Transwell Assay

A transwell assay was performed to estimate invasion ability. A498 and Caki-1 cells transfected with 40 nM miRNA mimics were collected, resuspended in serum-free medium, and reseeded at 1 × 10^5^ cells/well in a Matrigel-coated 24-transwell plate. The bottom wells were filled with a medium containing the 4× fetal bovine serum. The cells were washed once with PBS, fixed with methanol for 5 min, stained with 0.05% crystal violet solution (Sigma-Aldrich, Burlington, MA, USA) in 10% ethanol for 10 min, rinsed with distilled water, and dried after incubation for 72 h. The stained cells were then captured using Nikon Eclipse Ts2-FL Diascopic and Epi-fluorescence illumination model and counted.

### 2.8. Sphere-Forming Assay

A sphere-forming assay was performed to evaluate cancer-stem-cell-like characteristics. A498 and Caki-1 cells were seeded at a density of 3000 cells/well in a 6-well ultra-low attachment plate (Corning, NY, USA) or 100 cells/well in a 96-well ultra-low attachment plate (Corning) using Dulbecco’s Modified Eagle Medium/Nutrient Mixture F-12 medium supplemented with 2% B-27 (Gibco), 20 ng/mL epidermal growth factor (Gibco), and 20 ng/mL fibroblast growth factor b (Gibco). The cells were incubated until an increase of 50 μm of sphere diameter was observed and transfected with 100 nM miRNA mimics for 4 days. The diameter of the spheres was measured in a 6-well ultra-low attachment plate, and the number of spheres was counted in a 96-well ultra-low attachment plate after incubation.

### 2.9. Selection of Target Genes

To predict the target genes of miR-4284, DIANA-MICROT (http://www.diana.imis.athena-innovation.gr (accessed on 20 July 2022)), TargetScan (http://www.targetscan.org (accessed on 20 July 2022)), and miRDB (http://www.mirdb.org (accessed on 20 July 2022)) were used. Genes common to the top 100 genes in each of the 3 sites were estimated. The mRNA and protein expression levels of the genes were identified using qRT-PCR and Western blotting, respectively.

### 2.10. Western Blotting

A498 and Caki-1 cells were transfected with 40 nM miRNA mimics, collected with PBS, and lysed using PRO-PREP^TM^ Protein Extraction Solution (iNtRON). The lysates were quantified using the Pierce BCA Protein Assay Kit (Thermo Fisher Scientific) according to the manufacturer’s protocol. Fifteen micrograms of the sample was separated via 10% sodium dodecyl-sulfate polyacrylamide gel electrophoresis at 100 V for 90 min and transferred to a polyvinylidene fluoride membrane at 95 V for 120 min. The membranes were then blocked with 5% skim milk in Tris-buffered saline with Tween 20 for 1 h at room temperature (RT) and incubated with specific antibodies in a blocking buffer at 4 °C for 24 h. The primary antibodies used were as follows: anti-Cleaved-PARP (Cell signaling, Davers, MA, USA), anti-PARP (Cell signaling, Davers, MA, USA), anti-phospho-Rb (Cell signaling, Davers, MA, USA), anti-Rb (Cell signaling, Davers, MA, USA), anti-Cyclin D1 (Santa Cruz, Dallas, TX, USA), anti-PCNA (Santa Cruz, Dallas, TX, USA), anti-E-cadherin (Santa Cruz, Dallas, TX, USA), anti-N-cadherin (BD Biosciences, Franklin, NJ, USA), anti-ZO-1 (Cell signaling, Davers, MA, USA), anti-Fibronectin (Santa Cruz, Dallas, TX, USA), anti-RSBN1L (Bioss Antibodies, Boston, MA, USA), anti-GAD1 (Thermo Fisher Scientific), and anti-β-actin (Santa Cruz, Dallas, TX, USA). Next, the membranes were rinsed with TBST thrice every 10 min, incubated with goat anti-mouse IgG or goat anti-rabbit IgG in TBST for 2 h at RT, rinsed with TBST thrice every 10 min, and then detected using clarity Western-enhanced chemiluminescence substrate (Bio-Rad, Hercules, CA, USA) using a G:BOX Chemi XX6 gel doc system. The raw image for Western blot analyses is presented in Supplementary Figure S2. 

### 2.11. Reporter Assay

A reporter assay was performed to determine whether miR-4284 directly binds to the human GAD1 3′-UTR. The potential binding site of miR-4284 in the human GAD1 3′UTR was predicted using the miRNA target gene prediction program TargetScan (http://www.targetscan.org (accessed on 20 September 2022)) and cloned into a pGL3UC vector (provided by V.N. Kim, Seoul National University, Republic of Korea, Bioneer Inc.). A498 and Caki-1 cells were seeded into a 24-well plate at 70% confluence, incubated for 24 h, and co-transfected with cloned or empty vector (100 ng), Renilla vector (100 ng) and miR-4284 (40 nM) for 48 h using Lipofectamine 3000 (Thermo Fisher Scientific). Next, the cells were lysed using 1X Passive Lysis Buffer, and luciferase activity was measured using a Dual-Luciferase Reporter Assay System (Promega, Madison, WI, USA), according to the manufacturer’s protocol. Normalizing the value of the luciferase activity was calculated as follows: luciferase activity = firefly activity/Renilla activity.

### 2.12. In Vivo Xenograft Experiments

A xenograft experiment was performed to estimate the tumor-growth-suppressive function of miR-4284 in vivo. To establish the xenograft mice, Caki-1 cells were subcutaneously injected at 10^7^ cells in 100 μL PBS per mouse into the right leg of mice after 4-week-old female BALB/c nu/nu mice were adapted to their environment for 1 week. Once the tumor volume reached 200 mm^3^, tumors were transfected with miRNA mimics using in vivo-jetPEI (Polyplus-transfection, France), according to the manufacturer’s protocol. Briefly, 10 μg of miRNA mimics and 1.2 μL of in vivo-jet PEI were complexed, vortexed, and injected intratumorally at 50 μL per mouse after incubation for 15 min. The length of the tumor was measured twice a week using a Vernier caliper, and the volume of the tumor was calculated using the following equation: (volume of tumor) = (small length)^2^ × (large length) × (π/6). The mice were sacrificed when the tumors grew to 2500 mm^3^ and were collected for IHC analysis.

### 2.13. Immunohistochemistry Analysis

IHC was performed using a ready-to-use IHC/ICC kit (BioVision, Milpitas, CA, USA). After the tumor tissues were embedded in paraffin and sectioned, the tumor tissue section was deparaffinized, washed with distilled water, and incubated with protein-blocking solution for 5 min at 25 °C. Subsequently, the slides were incubated with specific primary antibodies, such as GAD1 (Thermo Fisher Scientific), PCNA (Santa Cruz, Dallas, TX, USA) and BAX (Santa Cruz), for 30 min at 25 °C. Next, the slides were incubated with horseradish peroxidase conjugated goat anti-mouse IgG or goat anti-rabbit IgG secondary antibodies for 30 min at 25 °C, stained with a 3,3′-diaminobenzidine reagent for 10 min at 25 °C, and captured using ZEISS Axioscan 7.

### 2.14. Overall Survival Analysis

Survival analysis was performed using the K–M plotter database (www.KMplot.com (accessed on 3 January 2023)) to investigate the correlation between OS and miR-4284 or GAD1 expression [17]. The settings of the parameters were as follows: (i) gene symbol, hsa-miR-4284 or GAD1; (ii) divided patients by auto-selecting the best cutoff; (iii) survival, OS; (iv) kidney renal clear cell carcinoma; and (v) grade, 2–4.

### 2.15. Statistical Analyses

Statistical analyses were performed using GraphPad Prism version 8 (GraphPad Software Inc., La Jolla, CA, USA). Statistical analyses of the data for OS analysis were performed using the K–M plotter (www.KMplot.com (accessed on 3 January 2023)). Pairwise comparisons were conducted to identify the significance between groups as follows: Mann–Whitney U test, Kruskal–Wallis test with Bonferroni post-hoc test, one-way analysis of variance test with Tukey’s post-hoc test, and Student’s *t*-test. Data were represented as mean ± standard error of the mean (error bars). Statistical significance was set as a *p*-value < 0.05.

## 3. Results

### 3.1. Overexpression of miR-4284 Inhibits Growth and Promotes Apoptosis in Renal Cancer Cells

**Figure 1:**
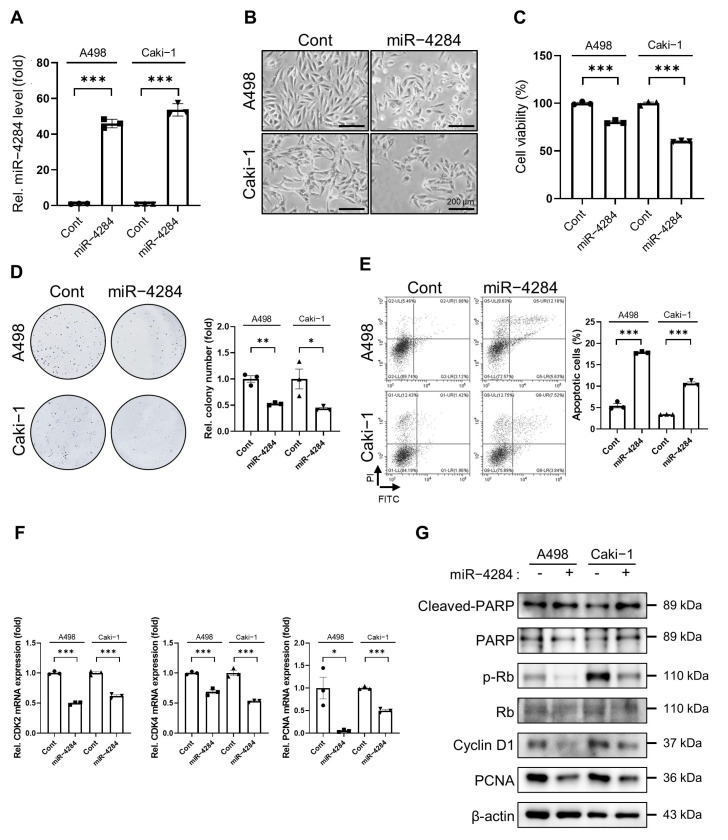
miR−4284 inhibits growth and induces apoptosis of renal cancer cells. (**A**) Identification of overexpression of miR−4284 level in renal cancer cells. A498 and Caki−1 cells were transfected with miRNA mimics for 48 h, and the miR−4284 level was examined using qRT-PCR analysis. Values were normalized by GAPDH expression. Data were analyzed using Student’s *t*-test and presented mean ± SEM. The *p*-value under 0.005 is indicated by ***. (**B**) Representative morphology of A498 and Caki−1 cells after transfection with miRNA mimics. The cells were transfected with miRNA mimics for 48 h and then captured using a microscope. (**C**) Cell viability measurement of miR−4284 in A498 and Caki−1 cells using CCK−8 assay. A498 and Caki−1 cells were transfected with miRNA mimics for 48 h, incubated with 10 μL of CCK−8 solution for 2 h, and then absorbance at 450 nm was measured. Data were analyzed using Student’s *t*-test and presented as mean ± SEM. The *p*−value under 0.005 is indicated by ***. (**D**) Images and graph of colony formation of A498 and Caki−1 cells following transfection with miRNA mimics. A498 and Caki−1 cells transfected with miRNA mimics were re-seeded in a 60 mm dish, cultured until colony forming, and images were captured. Data were analyzed using Student’s *t*-test and presented as mean ± SEM. The *p*-values under 0.05 and 0.01 are indicated by * and **, respectively. (**E**) Flow cytometry analysis of A498 and Caki−1 cells transfected with miRNA mimics to estimate apoptosis. A498 and Caki−1 cells were analyzed using FACS following transfection with miRNA mimics. Data were analyzed using Student’s *t*-test and presented as mean ± SEM. The *p*-value under 0.005 is indicated by ***. (**F**) mRNA expression of markers of proliferation in renal cancer cells using qRT-PCR analysis. A498 and Caki−1 cells transfected with miRNA mimic were prepared for total RNA isolation and qRT-PCR analysis was performed. Values were normalized by GAPDH expression. Data were analyzed using Student’s *t*-test and presented as mean ± SEM. The *p*-values under 0.05 and 0.005 are indicated by * and ***, respectively. (**G**) Protein expression analysis of markers of apoptosis and proliferation in renal cancer cells using Western blotting. A498 and Caki−1 cells were lysed for protein extraction following transfection with miRNA mimic, and Western blotting was performed.

Using the Gene Expression Omnibus database (GSE95385), we found that miR-4284 was more downregulated in patients with RCC compared with that in patients without RCC (Supplementary Figure S1). Therefore, we examined the tumor-growth-inhibiting functions of miR-4284 in renal cancer cells in vitro using the Cell Counting Kit (CCK)-8, colony formation, apoptosis assays, and quantitative reverse transcription-polymerase chain reaction (qRT-PCR). First, A498 and Caki-1 cells were transfected with miRNA mimics, and miR-4284 overexpression was determined using qRT-PCR (Figure 1A). The cell morphology and CCK-8 assay showed that the cells transfected with miR-4284 mimics exhibited lower viability compared with that observed in the cells transfected with negative miRNA mimics (Figure 1B,C). Furthermore, the number of colonies formed in the colony-formation assay decreased after the transfection with miR-4284 mimics (Figure 1D). The apoptosis assay revealed that the number of apoptotic cells significantly increased after the transfection with miR-4284 mimics (Figure 1E). Moreover, cell proliferation markers (cyclin-dependent kinase (CDK) 2, CDK4, and proliferating cell nuclear antigen (PCNA)) were downregulated after transfection with miR-4284 mimics (Figure 1F). In addition, Western blotting results showed that apoptosis markers (Cleaved-Poly-(ADP-ribose) polymerase [PARP] and PARP) increased, and proliferation markers (Phospho-retinoblastoma tumor suppressor protein [Rb], Rb, Cyclin D1, and PCNA) reduced following transfection with miR-4284 mimics (Figure 1G). These results indicated that miR-4284 overexpression suppressed proliferation and induced apoptosis in renal cancer cells.

### 3.2. Overexpressed miR-4284 Inhibits the Tumorigenic Characterization of Renal Cancer Cells

**Figure 2:**
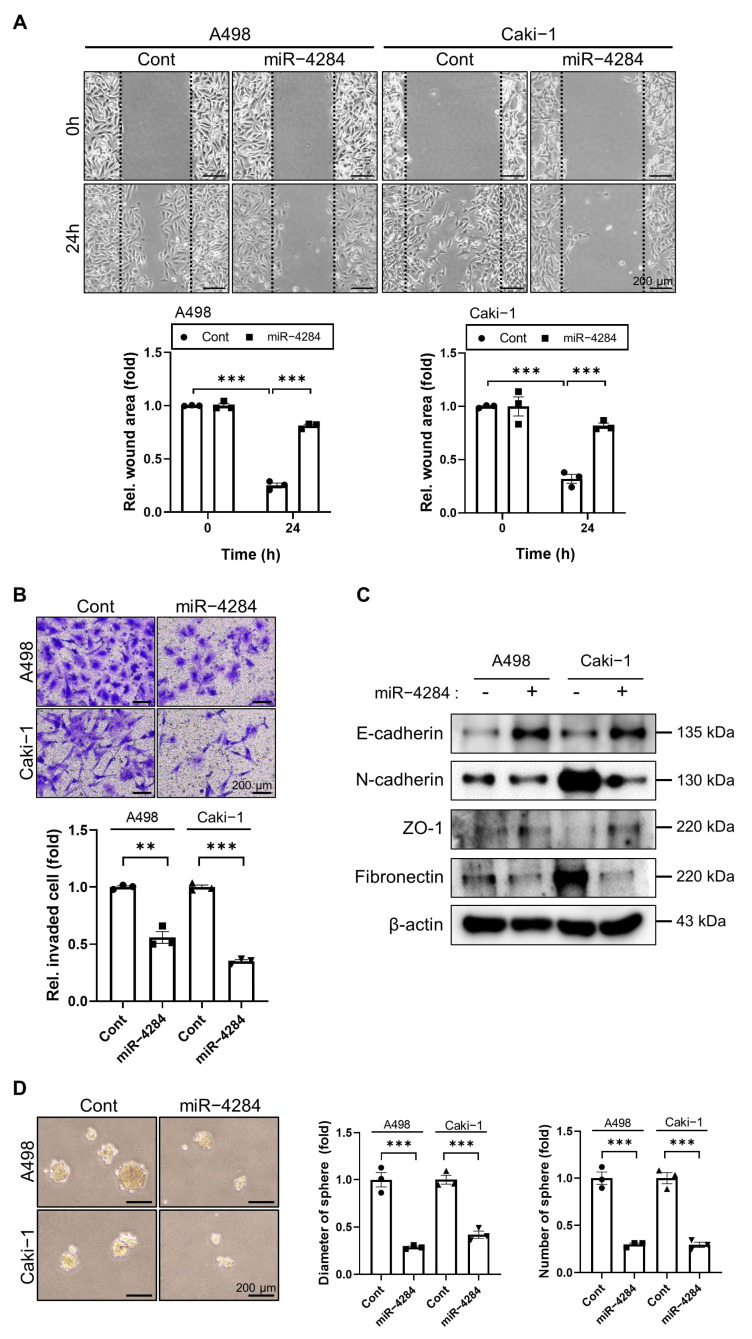
miR−4284 suppresses tumorigenic characterization of renal cancer cells. (**A**) Comparison of migrated cells following transfection with miRNA mimics using wound−healing assay in renal cancer cells. A498 and Caki−1 cells were transfected with miRNA mimics, scratched by a tip, incubated for 24 h, and images were captured using a microscope. Data were analyzed using Student’s *t*-test and presented as mean ± SEM. The *p*-value under 0.005 is indicated by ***. (**B**) Confirmation of invaded cells using transwell assay in renal cancer cells transfected with miRNA mimics. A498 and Caki−1 cells were transfected with miRNA mimics, re-seeded on a transwell coated with Matrigel, incubated for 72 h, and images were captured using a microscope. Data were analyzed using Student’s *t*-test and presented as mean ± SEM. The *p*-values under 0.01 and 0.005 are indicated by ** and ***, respectively. (**C**) Protein expression analysis of markers of migration in renal cancer cells transfected with miRNA mimic using Western blotting. (**D**) Representative images of spheres and graphs of diameter and the number of spheres using sphere formation assay following transfection with miRNA mimics. A498 and Caki−1 cells were cultured on an ultra-low attachment plate until 50 μm of sphere diameter, transfected with miRNA mimics, and images were captured using a microscope. Data were analyzed using Student’s *t*-test and presented as mean ± SEM. The *p*−value under 0.005 is indicated by ***.

To investigate the tumorigenic characteristics of renal cancer cells, we examined their migration, invasion, and cancer-stem-cell-like function using the wound-healing, transwell, and sphere-formation assays, respectively. The wound-healing assay showed that the wound area of miR-4284-mimic-transfected cells was larger than that of the negative miRNA-mimic-transfected cells (Figure 2A). Furthermore, A498 and Caki-1 cells transfected with miR-4284 mimics showed significantly reduced invasion on Matrigel-coated transwell plates (Figure 2B). In addition, epithelial markers (E-cadherin and zona occludens [ZO-1]) were upregulated, and mesenchymal markers (N-cadherin and Fibronectin) were downregulated in renal cancer cells transfected with miR-4284 mimics (Figure 2C). The sphere formation assay showed that the diameter and number of spheres significantly decreased upon the overexpression of miR-4284 mimics in renal cancer cells (Figure 2D). These results suggested that miR-4284 overexpression suppressed the tumorigenic features of renal cancer cells, including migration, invasion, and cancer-stem-cell-like ability.

### 3.3. Glutamate Decarboxylase 1 (GAD1) Is a Direct Target of miR-4284 in Renal Cancer Cells

**Figure 3:**
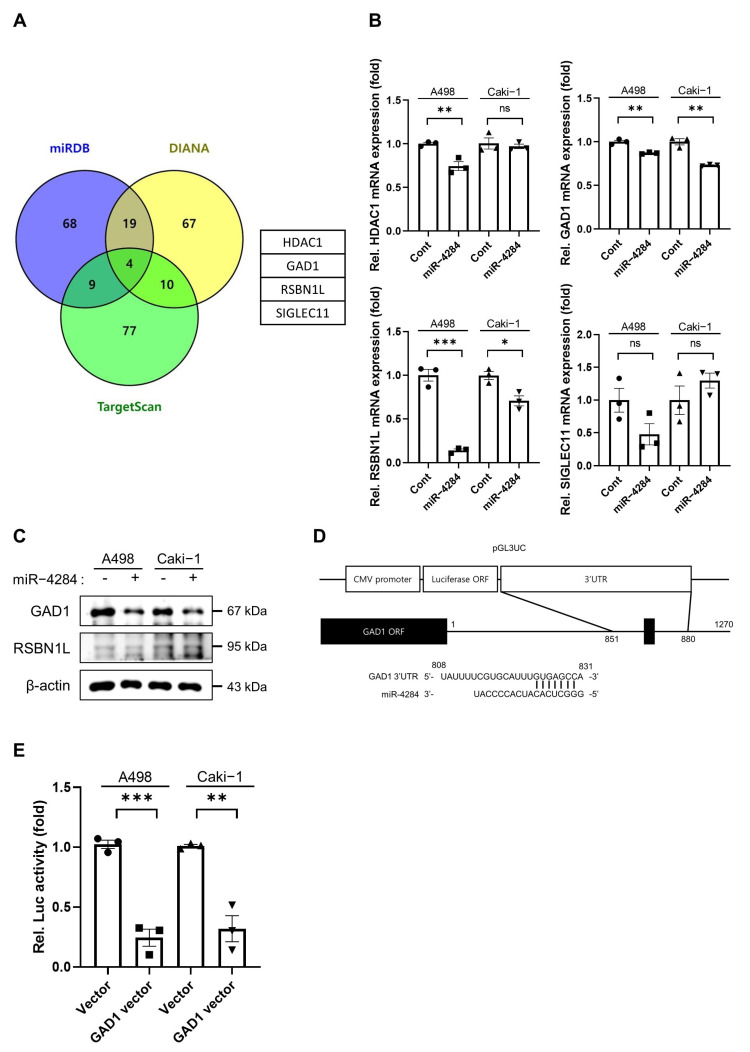
miR−4284 directly binds GAD1 as a target gene. (**A**) Venn diagram of putative target genes in the top 100 genes of three target gene prediction programs. (**B**) Validation of four putative target genes using qRT−PCR analysis. A498 and Caki-1 cells were transfected with miRNA mimic, isolated total RNA, and qRT−PCR analysis was performed. Values were normalized by GAPDH expression. Data were analyzed using Student’s *t*-test and presented as mean ± SEM. The *p*-values under 0.05, 0.01, and 0.005 are indicated by *, **, and ***, respectively. The ns means not significant. (**C**) Investigation of protein expression of two putative target genes using Western blot assay. A498 and Caki−1 cells were transfected with the miRNA mimic, lysed for preparing the protein, and a Western blot assay was performed. (**D**) Structure of a GAD1 cloned plasmid vector containing a DNA fragment of the predicted binding site of miR-4284 in the GAD1 3′−UTR. (**E**) Examination of the direct bind between miR−4284 and predicted binding site of miR−4284 in the GAD1 3′−UTR using a reporter assay. A498 and Caki−1 cells were co-transfected with empty/GAD1 cloned plasmid vector, Renilla vector, and miR−4284, and luciferase activity was measured. Data were analyzed using Student’s *t*-test and presented as mean ± SEM. The *p*-values under 0.01 and 0.005 are indicated by ** and ***, respectively.

Using miRNA target gene prediction programs, such as miRDB, TargetScan, and DIANA-MICROT, we explored the target genes of miR-4284 and identified four potential target genes: histone deacetylase 1, GAD1, round spermatid basic protein 1-like (RSBN1L), and sialic acid binding Ig-like lectin 11 (Figure 3A). Upon validation using qRT-PCR, only two genes (GAD1 and RSBN1L) were significantly downregulated (Figure 3B). Western blotting analysis showed that GAD1 protein expression was significantly reduced, whereas RSBN1L protein expression remained unchanged after transfection with miR-4284 mimics (Figure 3C). Therefore, we selected GAD1 as the target gene of miR-4284. To examine whether miR-4284 directly binds to the predicted binding site in the 3′-UTR of the GAD1 transcript, a GAD1-cloned plasmid vector containing the predicted binding site of miR-4284 in the 3′-UTR was generated for the reporter assay (Figure 3D). The reporter assay showed that the luciferase activity of A498 and Caki-1 cells co-transfected with the GAD1-cloned plasmid vector and the miR-4284 mimics was significantly lower than that of cells co-transfected with the empty plasmid vector and the miR-4284 mimics (Figure 3E). Overall, these results indicate that GAD1 is a direct target of miR-4284 in renal cancer cells.

### 3.4. Enhanced miR-4284 Level Suppresses Tumor Growth In Vivo

**Figure 4:**
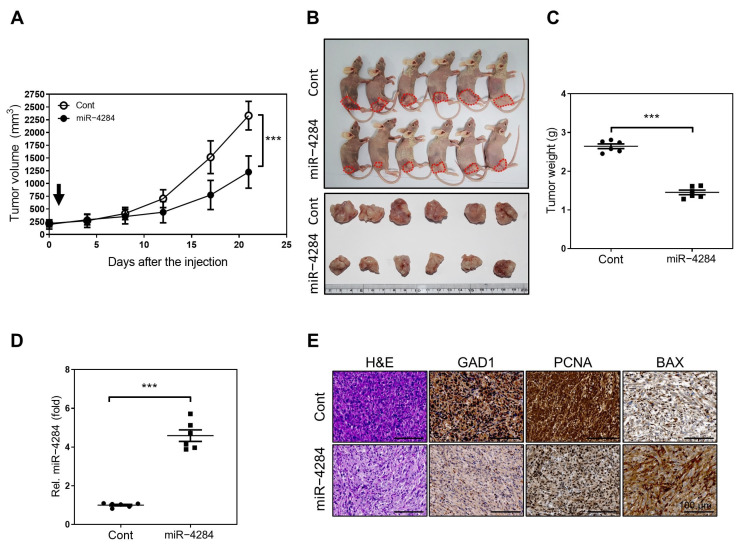
miR−4284 suppresses tumor growth in vivo. (**A**) Measurement of the growth rate of tumor volume at the indicated time after miRNA mimics injection. Data were analyzed using Student’s *t*-test and presented as mean ± SEM. The *p*−value under 0.005 is indicated by ***. (**B**) Images of xenograft models and resected tumors in mice at the endpoint after injection with miRNA mimics. The red circles is to indicate the tumor size. (**C**) Comparison with the weight between resected tumors of mice injected with negative miRNA mimics and mice injected with miR−4284 mimics. Data were analyzed using Student’s *t*-test and presented as mean ± SEM. The *p*−value under 0.005 is indicated ***. (**D**) Identification of enhanced miR−4284 in tumors of mice transfected with miRNA mimics using qRT−PCR analysis. Data were analyzed using Student’s *t*-test and presented as mean ± SEM. The *p*−value under 0.005 is indicated by ***. (**E**) Representative images of immunochemistry staining to GAD1, PCNA, and BAX proteins in resected tumor tissue.

The tumor-growth-inhibiting function of miR-4284 in renal cancer cells in vivo was estimated using a xenograft mouse model injected with Caki-1 cells. The tumor growth rate was suppressed upon transfection of the tumor with miR-4284 mimics. Furthermore, the volume of the tumors transfected with miR-4284 mimics was significantly smaller than that of the tumors transfected with negative miRNA mimics by the endpoint (Figure 4A–C). On the contrary, the miR-4284 expression level of the tumors transfected with miR-4284 mimics was significantly increased compared with that of tumors transfected with negative miRNA mimics (Figure 4D). In addition, an immunohistochemistry (IHC) analysis of the resected tumors showed that the expression of GAD1 as a target gene and PCNA as a marker of cell proliferation decreased, whereas the expression of Bcl-2-associated X (BAX), a pro-apoptotic marker, increased after injecting the miR-4284 mimic (Figure 4E). Overall, these results suggested that the increased miR-4284 expression suppressed the tumor growth in vivo.

### 3.5. miR-4284 or GAD1 Expression Level Correlates with the Overall Survival of Patients with Renal Cell Carcinoma

**Figure 5:**
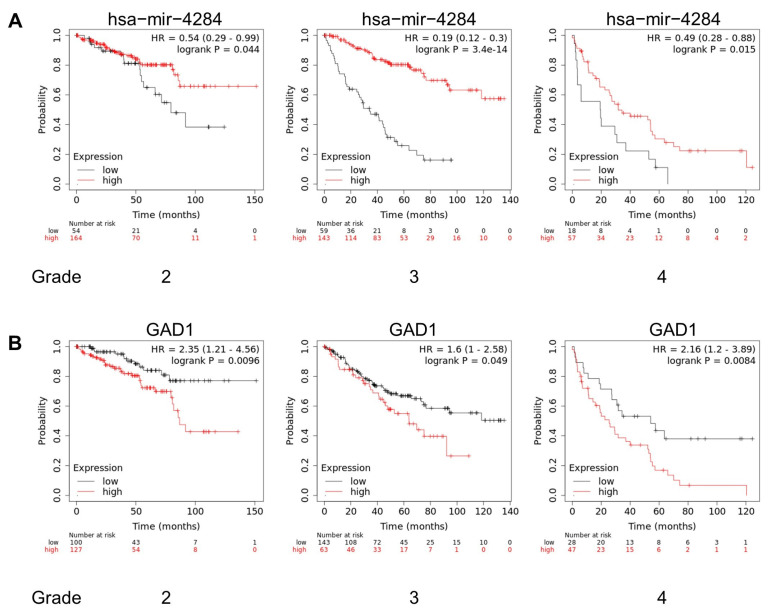
miR−4284 or GAD1 expression level correlates with the overall survival of patients with renal carcinoma. (**A**) Kaplan–Meier curves of correlation with miR−4284 expression level and patients with renal carcinoma according to cancer grade. (**B**) Kaplan–Meier curves of correlation with GAD1 expression level and patients with renal carcinoma according to cancer grade.

The overall survival (OS) of patients with RCC was analyzed using the Kaplan–Meier (K–M) plotter database to investigate the correlation between miR-4284 or GAD1 expression and the OS of patients with RCC. The patients with RCC showing high miR-4284 expression levels showed significantly higher OS than those with low miR-4284 expression levels across all tumor grades (Figure 5A). In contrast, the patients with low levels of GAD1, a target of miR-4284, showed significantly higher OS than those with high GAD1 expression levels across all grades (Figure 5B). These results indicated that miR-4284 and GAD1 could be used as diagnostic and prognostic markers in patients with RCC.

## 4. Discussion

RCC accounted for 431,288 new diagnoses and 179,368 deaths globally in 2020 [1]. Owing to recent advancements in diagnostic and treatment technologies, the early detection of cancer is increasing, and its mortality rate is rapidly decreasing. However, RCC is still detected, with 20–30% metastasis, and has a higher mortality rate than prostate or bladder cancer among urogenital cancers [18]. RCC is a heterogeneous malignancy with several genetic and acquired risk factors involved in its pathophysiology, including the VHL gene and protein polybromo-1 gene [19].

miRNAs affect cancer cell death, proliferation, migration, invasion, colony formation, and angiogenesis [4,5]. They act as both tumor suppressors and oncogenes by targeting proteins involved in various pathways [5]. Studies are being conducted to identify valuable miRNAs as targets in the pathogenesis, diagnosis, prognosis, and treatment of RCC [8,11,12,20,21]. However, studies on miR-4284 and RCC, which are associated with several cancers, are insufficient. Therefore, in this study, we aimed to determine the exact role of miR-4284 in RCC.

Munari et al. reported that miR-4284 was one of the downregulated miRNAs compared with normal renal tissue in an miRNA profiling study [20]. According to Zaravinos et al., chromophobe RCC and control had different expression levels of miR-4284, indicating that it might be a good discriminatory marker for identifying RCC subtypes [22]. Consistent with the results of previous studies, miR-4284 was downregulated in our study [19,21]. The two renal cell lines were transfected with miR-4284, which inhibited the proliferation and increased the apoptosis in renal cancer cells. In addition, it was confirmed that it is a factor involved in metastasis, as it has the ability to suppress cell migration and invasion in both A498 and Caki-1 cells. That is, miR-4284 plays an important role as a tumor suppressor, affecting cancer development, apoptosis, migration, invasion, and proliferation in RCC.

miR-4284 is a gene located on chromosome 7, and its most well-known target gene is tumor necrosis factor receptor-associated factor 4 (TRAF4) [23]. TRAF4 mediates tumor growth factor (TGF)-β-induced suppressor of mothers against decapentaplegic (SMAD) and non-SMAD signaling. In particular, in breast cancer, TRAF4 is a key determinant of breast cancer pathogenesis by regulating the TGF-β pathway [24]. In addition, miR-4284 directly inhibits the translation of genes, such as B-cell lymphoma/leukemia 10, histone deacetylase, homeobox A1, and Phosphatase and TENsin homolog deleted on chromosome 10, affecting apoptosis, differentiation, and proliferation in diffuse large B-cell lymphoma [23]. miR-4284 acts as an anti-tumor miRNA in colon cancer, which reduces perilipin 5 and inhibits epithelial-to-mesenchymal transition, leading to inhibited colon cancer tumorigenesis [15]. However, in this study, we confirmed that miR-4284 directly targeted GAD1, which differs from previously known target genes.

GAD1 is an important cofactor in the synthesis of gamma-aminobutyric acid (GABA), an inhibitory neurotransmitter [25]. In particular, GAD1 has been studied for its association with various cancers, including brain tumors, because of its important role in metabolism via the GABA shunt [26,27,28]. Schnepp et al. reported that GAD1 was upregulated in brain metastatic tumor cells and helped brain metastatic tumor cells adapt to the brain microenvironment by increasing GABA synthesis [28]. According to Yan et al., hypermethylation of the GAD1 promoter in colon and liver cancer cells increased GAD1 production [26]. In addition, an increased expression of GAD1 has been reported in nasopharyngeal, breast, and gastric cancers [26,29,30]. A recent study has demonstrated that GAD1, independent of the GABA shunt, influences the growth of cancer cells in lung cancer tissues by controlling amino acid homeostasis [31]. Based on our findings, miR-4284 is expected to affect the development and metastasis of renal cancer cells by directly targeting GAD1. However, further studies are required to clarify the effects of miR-4284 and GAD1 on renal cancer cells with or without a GABA shunt.

Animal experiments have confirmed that the growth of renal cancer cells is suppressed when the level of miR-4284 increases. In addition, in patients with RCC of all cancer stages, the higher the miR-4284 expression level and the lower the expression level of GAD1 (the target gene of miR-4284), the higher the OS. In other words, we confirmed that miR-4284 and GAD1 expression could be targets for prognostic factors and treatment in patients with RCC.

## 5. Conclusions

In conclusion, we demonstrated that miR-4284, which directly targets GAD1, affected tumor growth, metastasis, and apoptosis in RCC in vitro and tumor proliferation in vivo. Therefore, it is meaningful in that miR-4284 shows efficacy as a prognostic factor and has the potential role as a target for anticancer miRNA therapeutics in RCC.

## Figures and Tables

**Table 1:** Primers used in this study for qRT-PCR analysis.

Gene		Sequence (5′–3′)
CDK2	F	CCAGGAGTTACTTCTATGCCTGA
	R	TTCATCCAGGGGAGGTACAAC
CDK4	F	ATGGCTACCTCTCGATATGAGC
	R	CATTGGGGACTCTCACACTCT
PCNA	F	ACACTAAGGGCCGAAGATAACG
	R	ACAGCATCTCCAATATGGCTGA
HDAC1	F	CTACTACGACGGGGATGTTGG
	R	GAGTCATGCGGATTCGGTGAG
GAD1	F	GCTTCCGGCTAAGAACGGT
	R	TTGCGGACATAGTTGAGGAGT
RSBN1L	F	GCGGAGAGTGAACGGAGAAG
	R	GAGGGGCAAAGCTCCAAGAC
SIGLEC11	F	CTACTGCTGCTTATGGCTACTG
	R	CGAAAGAAGTACCATGCCTCATC
GAPDH	F	GGAGCGAGATCCCTCCAAAAT
	R	GGCTGTTGTCATACTTCTCATGG

## Data Availability

The authors confirm that the data supporting the findings of this study are available within the article and its Supplementary Materials.

## Supplementary Materials

The following supporting information can be downloaded at: https://www.mdpi.com/article/10.3390/cancers15153888/s1, Figure S1: miR-4284 was down regulated in patients with renal carcinoma from the GEO database (GSE95385). (A) Comparison of the miR-4284 expression level in patients with renal carcinoma compared to that in those without renal carcinoma. (B) Volcano plots of patients with renal car-cinoma vs. without renal carcinoma from the GSE95385. (C) Boxplot showing miRNA expression of each group following data normalization from the GES95385. (D) UMAP test showing miRNA separation from the GSE95385; Figure S2: Western blot bands of cell lysate in figure 3C.
