# Supplementary material for: miR-4284 Functions as a Tumor Suppressor in Renal Cell Carcinoma Cells by Targeting Glutamate Decarboxylase 1
Source: Cancers (Basel). 2023 Jul 30;15(15):3888. doi: 10.3390/cancers15153888 (PMC10417762; doi:10.3390/cancers15153888)
Supplement: Supplementary file 1 [file cancers-15-03888-s001.zip › cancers-2473880-supplementary.pdf]

cancers

MDPI

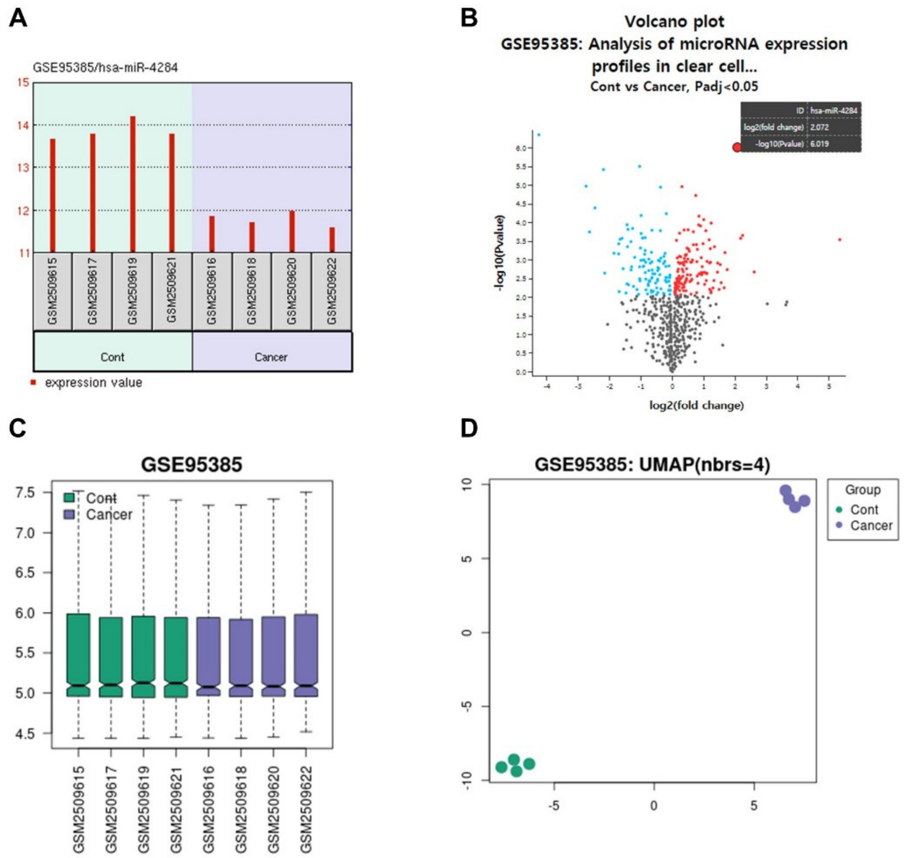

Supplementary Figure S1. miR-4284 was down regulated in patients with renal carcinoma from the GEO database (GSE95385). (A) Comparison of the miR-4284 expression level in patients with renal carcinoma compared to that in those without renal carcinoma. (B) Volcano plots of patients with renal carcinoma vs. without renal carcinoma from the GSE95385. (C) Boxplot showing miRNA expression of each group following data normalization from the GES95385. (D) UMAP test showing miRNA separation from the GSE95385.

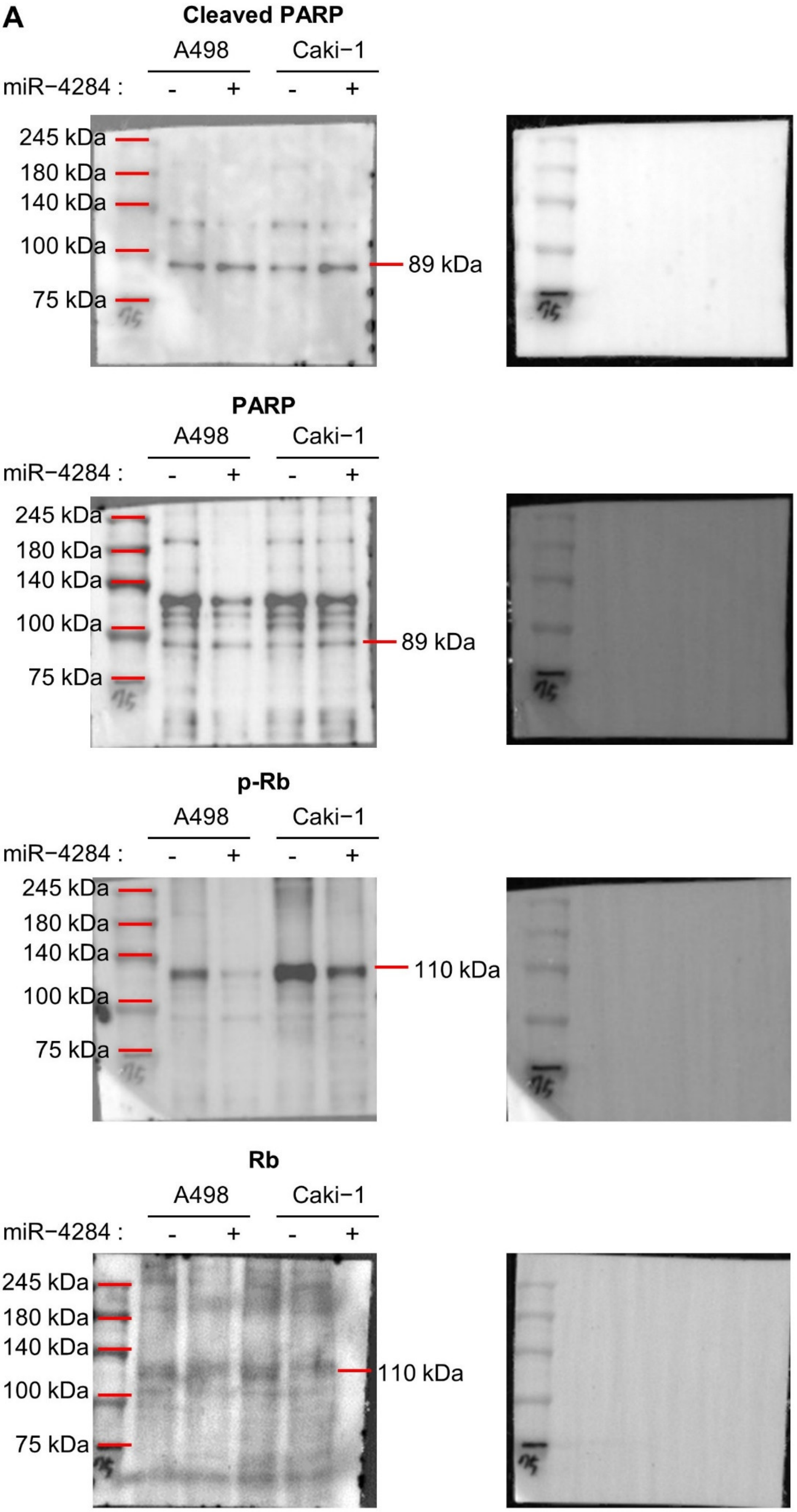
A
Cleaved PARP
A498
Caki−1
miR−4284 :
-
+
-
+
245 kDa
180 kDa
140 kDa
100 kDa
75 kDa
89 kDa
PARP
A498
Caki−1
miR−4284 :
-
+
-
+
245 kDa
180 kDa
140 kDa
100 kDa
75 kDa
89 kDa
p-Rb
A498
Caki−1
miR−4284 :
-
+
-
+
245 kDa
180 kDa
140 kDa
100 kDa
75 kDa
110 kDa
Rb
A498
Caki−1
miR−4284 :
-
+
-
+
245 kDa
180 kDa
140 kDa
100 kDa
75 kDa
110 kDa

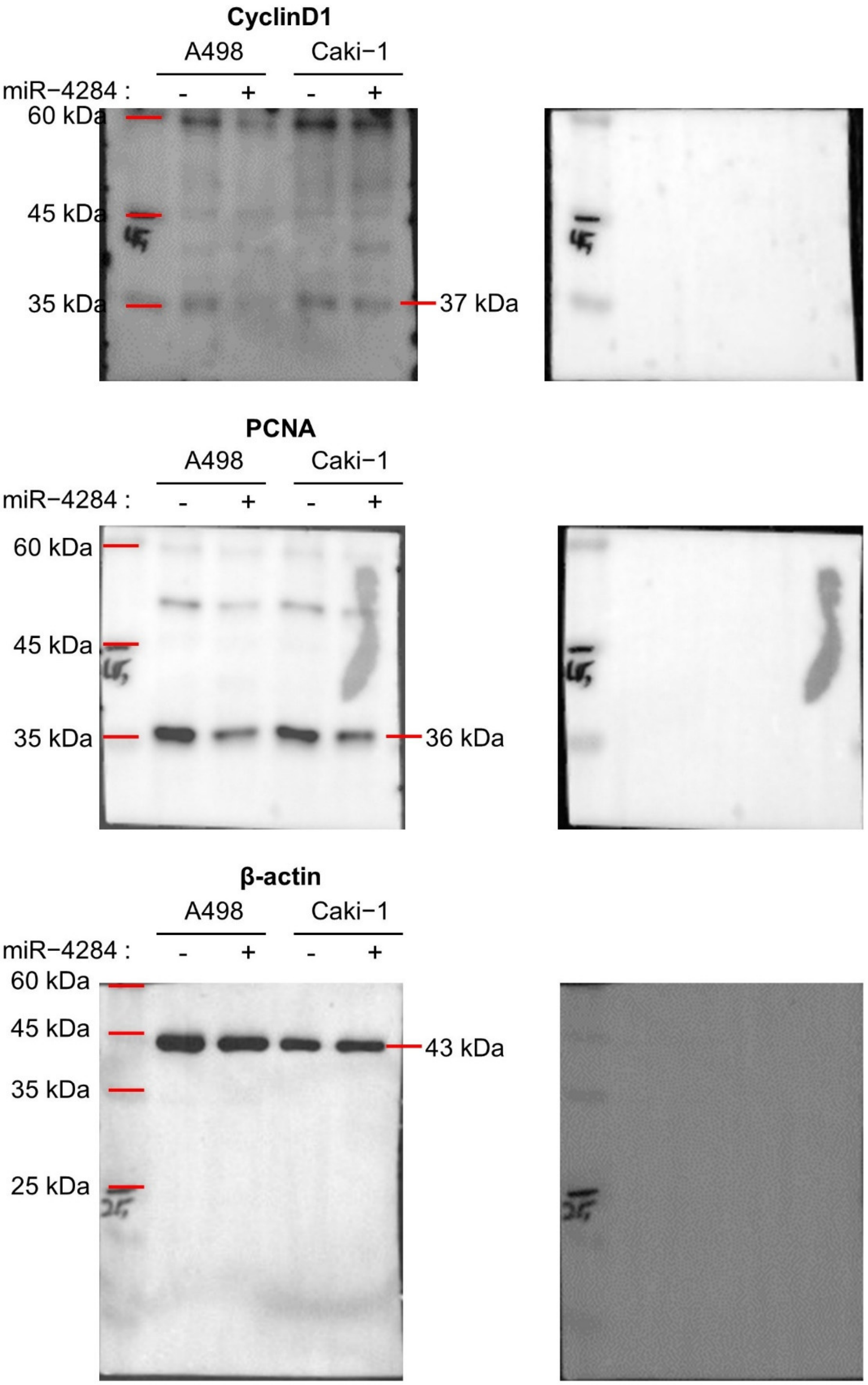
CyclinD1
A498
Caki−1
miR−4284 : - + - +
60 kDa
45 kDa
35 kDa
37 kDa
PCNA
A498
Caki−1
miR−4284 : - + - +
60 kDa
45 kDa
35 kDa
36 kDa
β-actin
A498
Caki−1
miR−4284 : - + - +
60 kDa
45 kDa
35 kDa
25 kDa
43 kDa

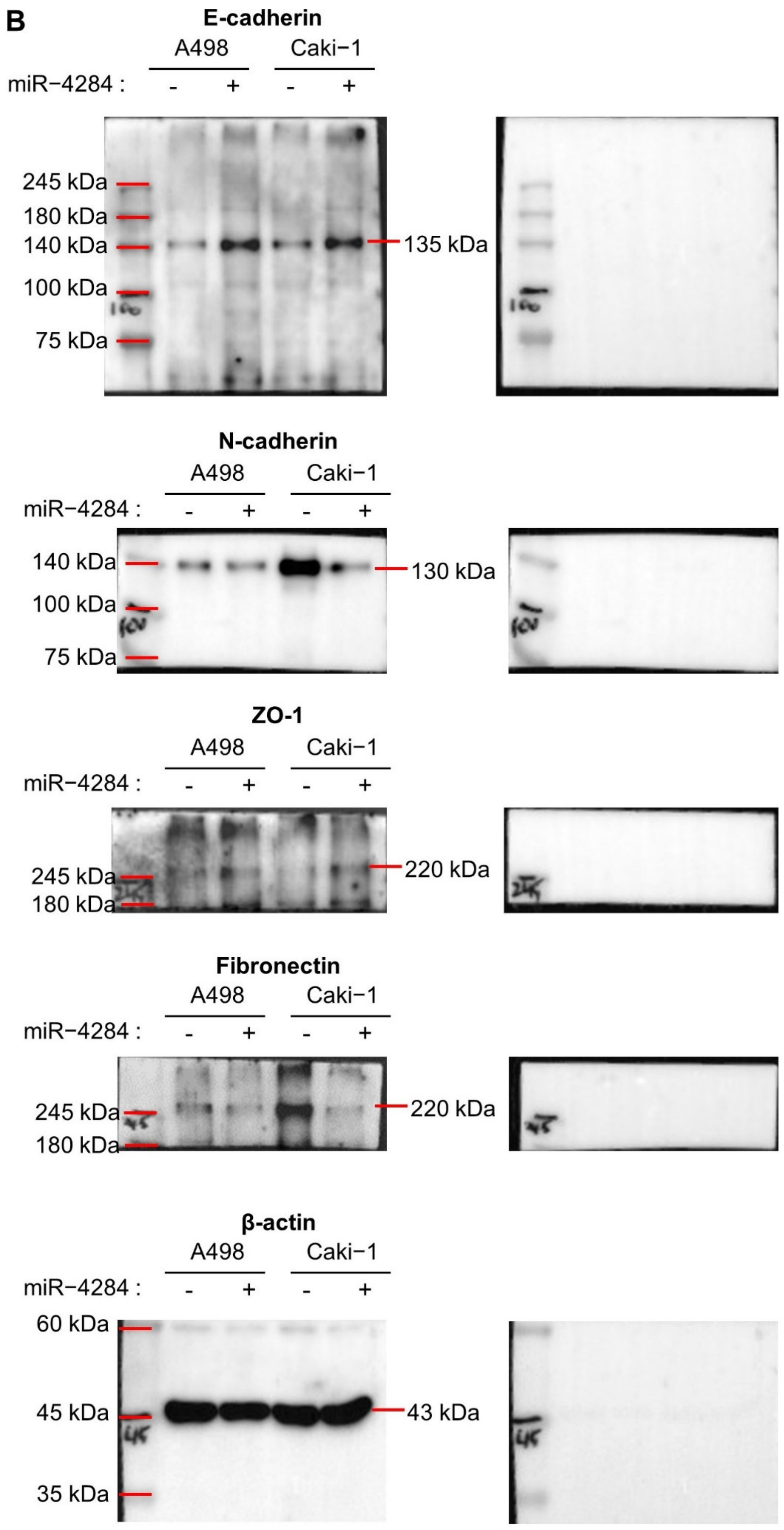
B
E-cadherin
A498
Caki−1
miR−4284 :
- + - +
245 kDa
180 kDa
140 kDa
100 kDa
75 kDa
135 kDa
N-cadherin
A498
Caki−1
miR−4284 :
- + - +
140 kDa
100 kDa
75 kDa
130 kDa
ZO-1
A498
Caki−1
miR−4284 :
- + - +
245 kDa
180 kDa
220 kDa
Fibronectin
A498
Caki−1
miR−4284 :
- + - +
245 kDa
180 kDa
220 kDa
β-actin
A498
Caki−1
miR−4284 :
- + - +
60 kDa
45 kDa
35 kDa
43 kDa

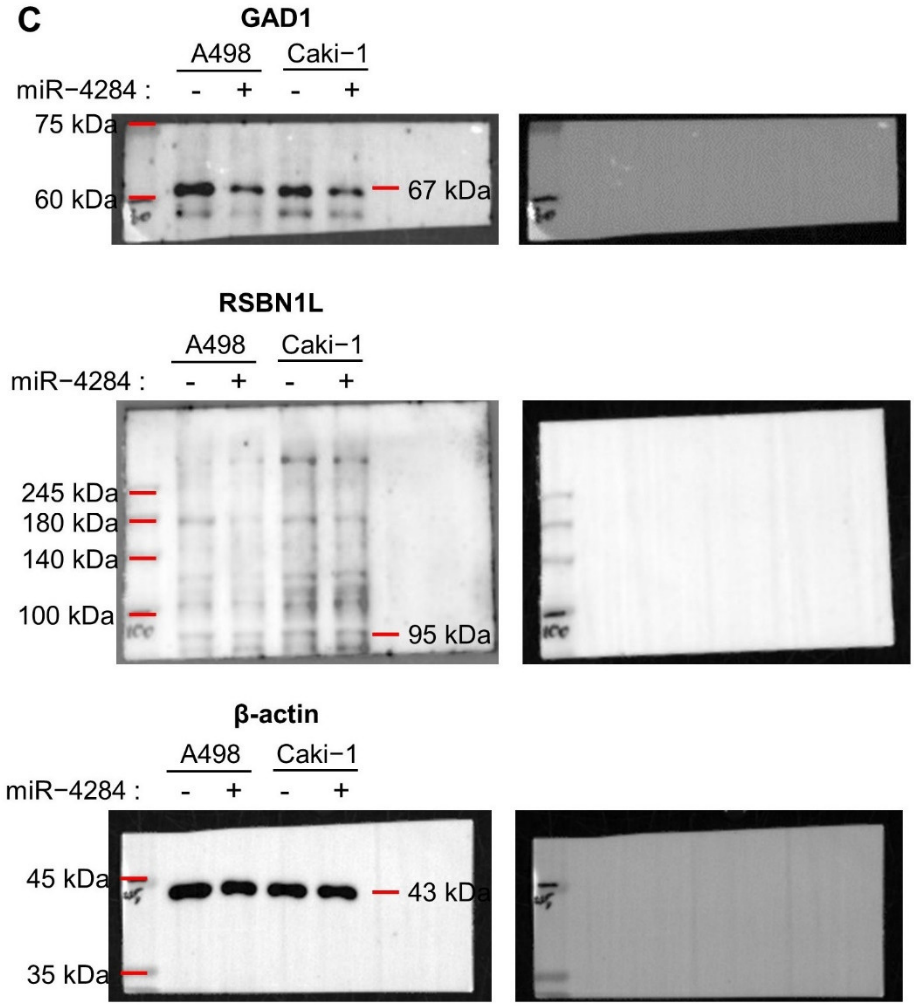

Supplementary Figure S2. Western blot of cell lysate in figure. (A) Western blot bands of figure 1G. (B) Western blot bands of figure 2C. (C) Western blot bands of figure 3C.
